# Integrating Systemic Inflammation and Longitudinal Weight Trajectories Improves Prognostic Stratification in Gastrointestinal Cancers: A Real-World Retrospective Study

**DOI:** 10.3390/cancers18172808

**Published:** 2026-08-29

**Authors:** Saunjoo L. Yoon, Jung A Kim, Oliver Grundmann, Debra Lynch-Kelly, Thomas J. George

**Affiliations:** 1 Center for Palliative Care Research and Education, Department of Biobehavioral Nursing Science, College of Nursing, University of Florida, Gainesville, FL 32610, USA; 2 College of Nursing, Hanyang University, Seoul 04763, Republic of Korea; joyhippo@hanyang.ac.kr; 3 College of Pharmacy, University of Florida, Gainesville, FL 32610, USA; grundman@ufl.edu; 4 Loewenberg College of Nursing, University of Memphis, Memphis, TN 38152, USA; dlkelly4@memphis.edu; 5 Division of Hematology & Oncology, Department of Medicine, College of Medicine, University of Florida, Gainesville, FL 32610, USA; thom.george@medicine.ufl.edu

**Keywords:** overall survival, unintentional weight loss, gastrointestinal cancer, biomarkers, High-Sensitivity modified Glasgow Prognostic Score (hs-mGPS), body mass index, longitudinal weight change

## Abstract

**Background/Objectives**: This study examined whether the high-sensitivity modified Glasgow Prognostic Score (hs-mGPS) and daily weight change by percentile (DWtCP) are associated with overall survival in patients with gastrointestinal (GI) cancers, overall and across BMI categories. **Methods:** We analyzed de-identified electronic health record data from 176 patients with GI cancers who had albumin, high-sensitivity C-reactive protein (hs-CRP), and longitudinal weight measurements. hs-mGPS was calculated from baseline albumin and hs-CRP. DWtCP, derived from serial weight measurements, was categorized as weight gain, moderate weight loss, or severe weight loss. Survival was evaluated using Kaplan–Meier and Cox proportional hazards models. **Results:** Nearly 70% of patients had hs-mGPS = 2, which was associated with poorer survival in the earlier part of follow-up (Breslow *p* = 0.011; log-rank *p* = 0.062), a pattern also observed in the high-BMI group (Breslow *p* = 0.045; log-rank *p* = 0.059). DWtCP was strongly associated with survival (log-rank *p* < 0.001; Breslow *p* < 0.001). Patients with moderate weight loss had the most favorable 1200-day Kaplan–Meier survival estimate (54.6%), compared with 5.1% for severe weight loss and 10.9% for weight gain. In multivariable analysis, moderate weight loss was associated with substantially lower mortality risk than weight gain (HR = 0.194, *p* < 0.001). In BMI-stratified models, DWtCP and primary cancer site remained independent predictors, whereas albumin and hs-CRP did not reach statistical individual significance, supporting the value of the composite hs-mGPS score. **Conclusions:** In this real-world retrospective cohort, hs-mGPS and DWtCP provided complementary prognostic information. Routine monitoring of systemic inflammation and longitudinal weight trajectories may enable pragmatic risk stratification and early identification of high-risk patients, particularly those with elevated BMI.

## Simple Summary

This retrospective study of 176 patients with gastrointestinal cancers evaluated whether integrating systemic inflammation and longitudinal weight change improves prognostic stratification. The high-sensitivity modified Glasgow Prognostic Score (hs-mGPS), derived from baseline albumin and hs-CRP, and daily weight change by percentile (DWtCP), derived from serial weights, were examined overall and across BMI categories. Nearly 70% of patients had hs-mGPS = 2, which was associated with poorer survival, most clearly in the earlier part of follow-up and among high-BMI patients. DWtCP was strongly associated with survival; patients with moderate weight loss had the most favorable 1200-day survival, outperforming both severe weight loss and weight gain groups. In multivariable and BMI-stratified models, DWtCP and primary cancer site were independent predictors of mortality, whereas albumin and hs-CRP contributed through the composite hs-mGPS score rather than individually. These findings suggest that routine monitoring of hs-mGPS and weight trajectories using electronic health record data may enable pragmatic risk stratification in GI cancers, especially in patients with higher BMI.

## 1. Introduction

Gastrointestinal (GI) cancers accounted for the second-highest number of new cases (17.5%) and the highest number of deaths (28.5%) among all cancers in 2026 [1]. Cancer-associated cachexia, a multifactorial syndrome characterized by involuntary weight loss and reduced skeletal muscle mass (with or without loss of fat mass) [2] that cannot be fully reversed by conventional nutritional support and leads to progressive functional impairment, is more prevalent in this population than in other cancer types and is strongly associated with increased mortality [3,4] and significantly impacts survival [5]. According to international consensus criteria, cancer cachexia is diagnosed when there is weight loss greater than 5% over the past 6 months or weight loss greater than 2% in individuals with BMI < 20 kg/m^2^ or evidence of sarcopenia [2]. The Global Leadership Initiative on Malnutrition (GLIM) further defines cancer cachexia as disease-related malnutrition with inflammation, integrating phenotypic features (weight loss, low BMI, reduced muscle mass) and etiologic criteria (reduced intake/assimilation and systemic inflammation) [6]. Despite these widely accepted definitions, cachexia remains underrecognized and undertreated in routine practice, in part due to the lack of simple, routinely available tools for early risk stratification, which is supported by the recent survey study [7].

Baseline body mass index (BMI) and weight loss have long been recognized as important prognostic factors in cancer. Prior studies indicated that weight loss rates were similar across baseline BMI categories, yet patients with low BMI demonstrated significantly worse survival than those with normal and high BMI [5]. In parallel, baseline metabolic and systemic inflammatory biomarkers are linked to a significantly increased risk of cancer mortality [8]. However, the relationship between survival across BMI categories and changes in key biomarkers, particularly albumin and C-reactive protein (CRP), remains insufficiently characterized in GI cancers. Moreover, most studies have not operationalized weight loss in a way that directly maps onto consensus cachexia thresholds, limiting the clinical interpretability of findings.

The modified Glasgow Prognostic Score (mGPS), derived from C-reactive protein (CRP) and albumin levels, is an established prognostic indicator of disease progression and survival in cancer patients [9,10,11]. The high-sensitivity mGPS (hs-mGPS), which incorporates high-sensitivity CRP (hs-CRP), has demonstrated improved prognostic performance compared with the traditional m-GPS (HR: 5.77 vs. 2.6; *p* < 0.001; AUC: 0.734 vs. 0.695) [12]. Higher hs-mGPS is consistently associated with poorer survival outcomes in cancer patients [13]. However, the prognostic utility of hs-mGPS across BMI categories has not been sufficiently established in patients with GI cancers. Furthermore, the impact of post-diagnosis weight changes on survival in this population remains unclear.

Longitudinal weight change may capture dynamic aspects of nutritional and metabolic status that are not reflected in a single baseline BMI measurement. Yet, few studies have integrated inflammation-based scores with serial weight data to examine their joint prognostic value in relation to consensus cachexia criteria. To our knowledge, this study is among the first to evaluate the combined prognostic utility of hs-mGPS and longitudinal weight change, stratified by BMI, in a real-world GI cancer cohort. The purpose of this study was to examine the associations among inflammatory biomarkers (albumin and hs-CRP), BMI categories, and overall survival (OS) in patients with GI cancers. Specifically, the study aimed to (1) evaluate the predictive value of the hs-mGPS for OS and (2) determine the prognostic impact of weight change and BMI on OS, both overall and stratified by BMI group. Unlike prior studies that have examined biomarkers or body weight separately, our analysis integrates both dimensions using real-world electronic health record data, thereby capturing a more clinically meaningful picture of nutritional and inflammatory risk.

## 2. Materials and Methods

### 2.1. Study Design

This retrospective comparative correlational study used de-identified data from the Integrated Data Repository (IDR) at the Clinical Translational Science Institute (CTSI) at the University of Florida. Institutional Review Board (IRB) approval was obtained prior to data access (IRB # 201400215).

### 2.2. Sample

**Figure 1:**
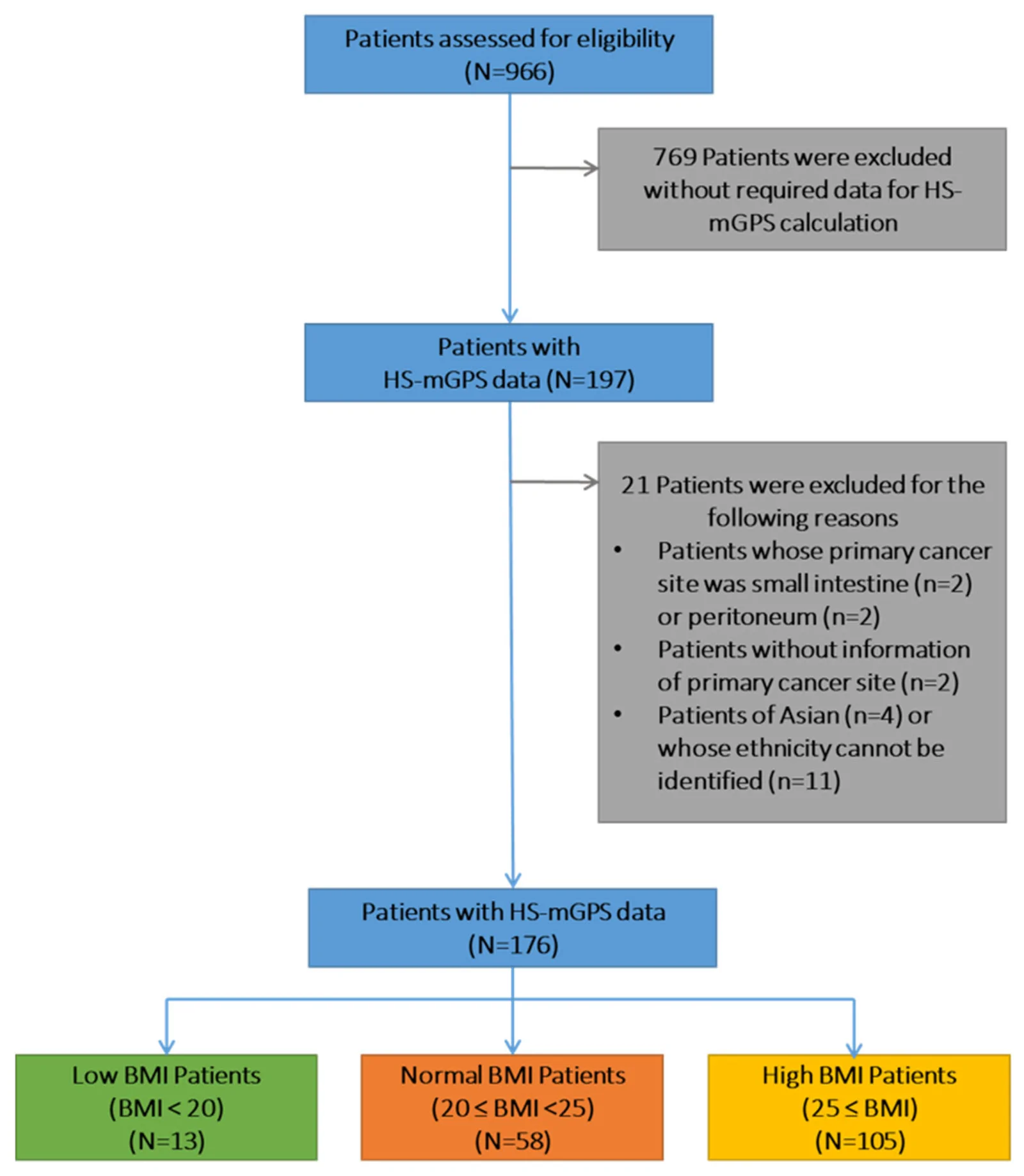
Flowchart of the sample selection process. Of 966 patients with gastrointestinal (GI) cancers identified in the source dataset, 197 met the eligibility criteria, and 176 constituted the final analytic sample.

Of the 966 patients with GI cancers in the original dataset, 197 met initial eligibility criteria. Inclusion criteria were patients who visited the institution between June 2011 and March 2014 with (1) age 21 years or older at the time of visit, (2) diagnosis of any GI cancers as the primary sites (e.g., gastric, biliary, esophageal, colorectal, pancreatic, small intestinal cancers) [ICD-9 codes: 150–159], (3) at least two recorded weight measurements, and (4) availability of both albumin and hs-CRP laboratory results. Data from 21 patients were excluded due to incomplete information, resulting in a final analytic sample of 176 patients (Figure 1).

### 2.3. Data Acquisition and Ethical Considerations

Data were extracted from the institution’s IDR, which integrates electronic health record data through the Informatics for Integrating Biology and the Bedside (i2b2) platform to support clinical and translational research. Approval was obtained from the IRB (#201400215) at the study site prior to de-identified data extraction. The research team members had no direct patient contact. All data were kept secure on encrypted servers within the academic Health Science Center network.

### 2.4. Variables of Interest

The following variables were extracted from the data:

1. Demographic information, including age, sex, and race/ethnicity.
2. Clinical characteristics: GI cancer type (ICD-9 codes from 150 to 159) with confirmed cancer stage.
3. Weight measurements: longitudinal weight data from the first recorded value through the last available measurement during the study period; time intervals were calculated in days from each patient’s first recorded observation in the institutional electronic health record, which defined that patient’s index date (Day 0) and entry into the cohort.
4. Baseline body mass index (BMI): calculated at the first observation point.
5. Laboratory values: the first albumin and hs-CRP values recorded after cohort entry (referred to as baseline values).
6. Overall survival was defined as the number of days from the index date to death from any cause; patients not known to have died were censored at the date of their last recorded observation. Because the de-identified data did not include the date of cancer diagnosis, and some patients had been diagnosed elsewhere before their first visit to the study institution, time zero was cohort entry rather than diagnosis. The first albumin and hs-CRP values were recorded a median of 94 days (IQR 23–378) and 96 days (IQR 21–383) after the index date, respectively, and the first weight measurement was recorded a median of 5 days (IQR 1–31) after the index date. Cancer stage could not be determined from the de-identified records for 47 patients (26.7%) and was unrecorded for 3 (1.7%); these patients were retained under an explicit “unknown” category in all analyses rather than excluded, and no imputation was performed. Availability of albumin, hs-CRP, and at least two weight measurements was an inclusion criterion, so these variables were complete by design.

### 2.5. Measurements

**High-sensitivity modified GPS (hs-mGPS):** hs-mGPS incorporates high-sensitivity C-reactive protein (hs-CRP) with a lower cut-off value (3 mg/L) compared with the traditional CRP threshold (10 mg/L) used in mGPS [12]. The Modified Glasgow Prognostic Score (mGPS) is a validated systemic inflammation-based scoring system using C-reactive protein and albumin measurements [0 = CRP < 10 mg/L and albumin ≥ 3.5 g/dL; 1 = CRP > 10 mg/L; 2 = CRP > 10 mg/L and albumin < 3.5 g/dL] [9], which originated from the Glasgow Prognostic Score (GPS) to predict overall and cancer-specific survival [10,14]. Hs-mGPS is reliable [13] and validated for enhanced prognostic value compared to mGPS [12,15,16], and its scores are defined as 0 [hs-CRP < 3 mg/L and albumin > 3.5 g/dL], 1 [hs-CRP > 3 mg/L and albumin ≥ 3.5 g/dL], and 2 [hs-CRP > 3 mg/L and albumin < 3.5 g/dL] (Table 1) [12,15,16].

**Weight changes:** To account for variability in follow-up duration, daily weight change by percentile (DWtCP) was calculated as the percent change in body weight from baseline to last observation divided by the number of days between the two measurements:

$$\frac{\left(\mathrm{last}\;\mathrm{weight}-\mathrm{baseline}\;\mathrm{weight}\right)/\mathrm{baseline}\;\mathrm{weight}}{\mathrm{days}\;\mathrm{between}\;\mathrm{measurements}}\times 100.$$

Then, DWtCP was categorized into three groups based on the magnitude and direction of weight change over time:

Weight gain: DWtCP > 0.00% per day.

Moderate weight loss: −0.03% ≤ DWtCP ≤ 0.00% per day.

Severe weight loss: DWtCP < −0.03% per day.

In this metric, positive values indicate weight gain, and negative values indicate weight loss. The cut-off point of −0.03% per day was selected to distinguish more pronounced, clinically meaningful weight decline from more modest changes over the observation period. This threshold corresponds to approximately 1.1% weight loss per month (about 13% per year) and was chosen to separate patients with substantial ongoing weight loss from those with relatively stable or modestly declining weight.

**Body mass index (BMI):** Patients were categorized into three BMI groups: low BMI (<20 kg/m^2^), normal BMI (20 to <25 kg/m^2^), and high BMI (≥25 kg/m^2^).

### 2.6. Statistical Analysis

Statistical analyses for this study were performed using SPSS 27.0 (IBM Corporation, Armonk, NY, USA, 2020) and Microsoft Excel Office Professional Plus 2016 (Microsoft Corporation, Redmond, WA, USA, 2016). Categorical variables were summarized using frequencies and percentages, and continuous variables using means ± SD or medians with IQRs, as appropriate to their distribution. Descriptive statistics were performed for demographic characteristics and clinicopathologic characteristics across the three BMI groups.

Due to the small sample size in the low BMI group (n = 13), this group was excluded from comparative analyses, and comparisons were limited to two BMI groups (normal vs. high). Differences in categorical variables were assessed using the chi-square test and continuous variables using the Jonckheere–Terpstra test.

Overall survival (OS) was analyzed using the Kaplan–Meier method, with the log-rank and Breslow tests to evaluate associations with hs-mGPS and DWtCP categories. The Cox proportional hazards regression models were used for univariate and multivariate analyses to identify prognostic factors for OS in the overall sample and within BMI strata (normal and high). Variables associated with OS at a two-sided *p* < 0.10 in univariate analysis were entered into the multivariate models. A more liberal threshold than the conventional *p* < 0.05 was applied at the screening stage, as selection of 0.05 has been shown to exclude variables that prove important once confounding is accounted for [17,18,19]. Hazard ratios (HRs) and 95% confidence intervals (CIs) are reported. The proportional hazards assumption was assessed for each covariate using scaled Schoenfeld residuals. All tests were two-sided, and *p* < 0.05 was considered statistically significant.

## 3. Results

### 3.1. Patient Characteristics

The characteristics of patients and their distribution across BMI groups are presented in Table 2. The mean age of patients was 65.2 ± 12.3 years (range: 29–92), with 53.4% aged ≥ 65 years. The majority of patients were White (82.4%) and male (59.1%). Approximately 40% of patients were diagnosed with anemia at baseline. The most common primary cancer sites were colorectal (29%) and pancreatic (29%) cancers, followed by hepatobiliary cancers (25%). Cancer stage was unassigned in 26.7% of patients, followed by stages 2 and 4, which were most frequent (both 21.6%).

Baseline biomarker profiles reflected a high inflammatory burden. The median albumin level was 3.0 g/dL (IQR: 2.44–3.60; reference: 3.5–5.0 g/dL), and the median hs-CRP was 70.20 mg/L (IQR: 23.80–163.70; reference: <3.0 mg/L). Nearly 70% of patients had both elevated hs-CRP and decreased albumin levels, corresponding to an hs-mGPS score of 2.

Weight change patterns differed across BMI groups, although the difference did not reach statistical significance (*p* = 0.073). In the normal BMI group, 44.8% of patients had moderate weight loss, whereas 45.7% of patients in the high BMI group had severe weight loss. Hs-CRP levels differed significantly between BMI groups (*p* = 0.014), with higher levels in the high BMI group (Table 2).

**Figure 2:**
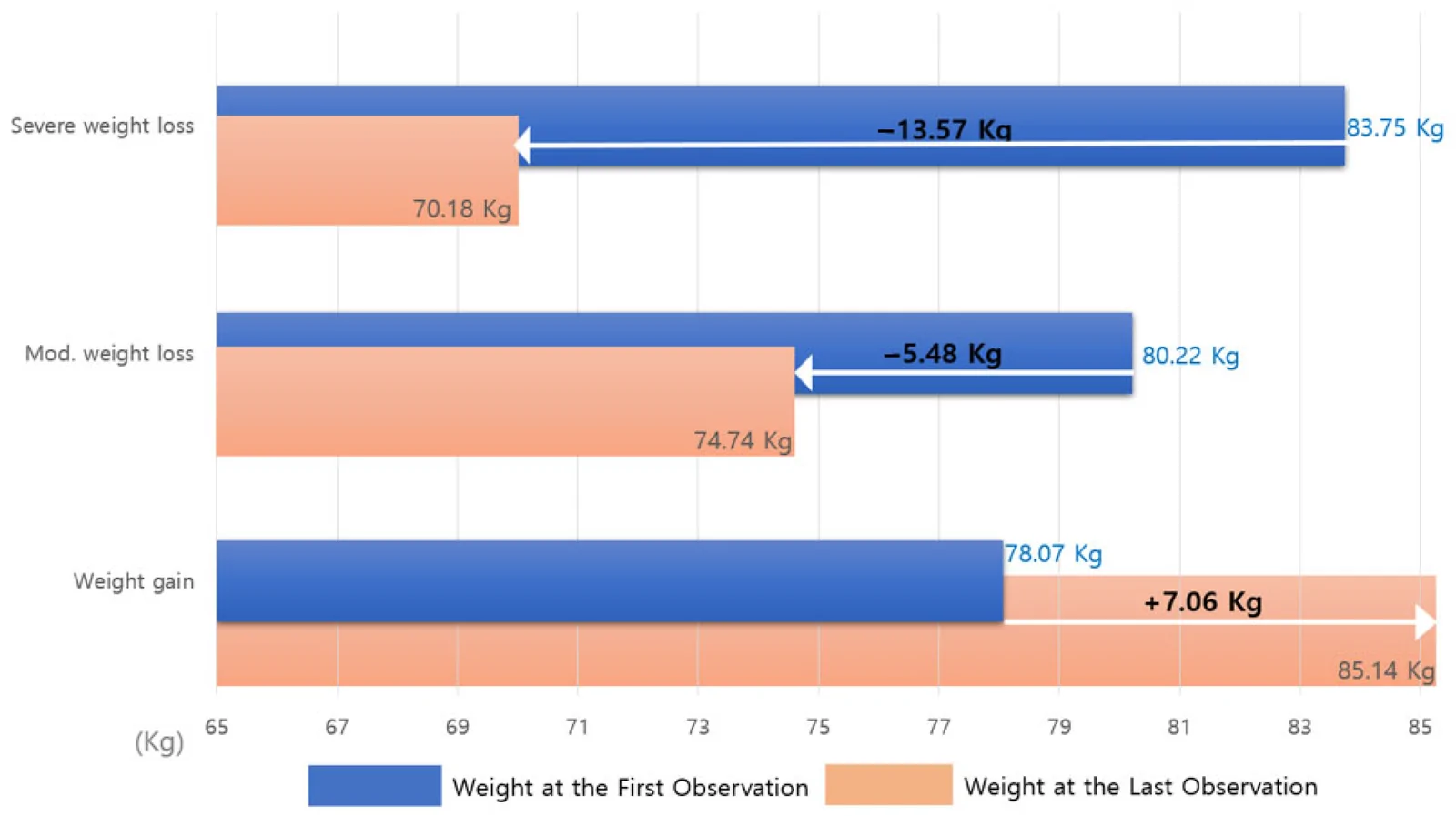
Mean body weight (kg) at the first and last observations across DWtCP groups. DWtCP, daily weight change by percentile: weight gain (DWtCP > 0.00), moderate weight loss (−0.03 ≤ DWtCP ≤ 0.00), and severe weight loss (DWtCP < −0.03% per day).

At diagnosis, 59.7% of patients were classified as having a high BMI. During the observation period, 76.7% experienced weight loss. Mean body weight decreased from 83.17 ± 19.50 kg at baseline to 77.29 ± 19.60 kg at last observation. The mean absolute weight change was −0.028 ± 1.032 kg/day, corresponding to a mean DWtCP of −0.025 ± 1.177% (range: −12.03 to 5.85). Of all patients (N = 176), 38.1% had moderate weight loss (mean loss: 5.48 ± 5.46 kg; 6.8% of body weight), and 38.6% had severe weight loss (mean loss: 13.57 ± 11.11 kg; 16.2% of body weight) (Figure 2).

### 3.2. Association Between hs-mGPS and Overall Survival (OS)

Survival analyses included the 163 patients in the normal and high BMI groups. During the observation period, 62 deaths (38.0%) occurred, and 101 patients (62.0%) were censored at their last recorded observation; the median observation time was 292 days, with a maximum of 1013 days.

**Figure 3:**
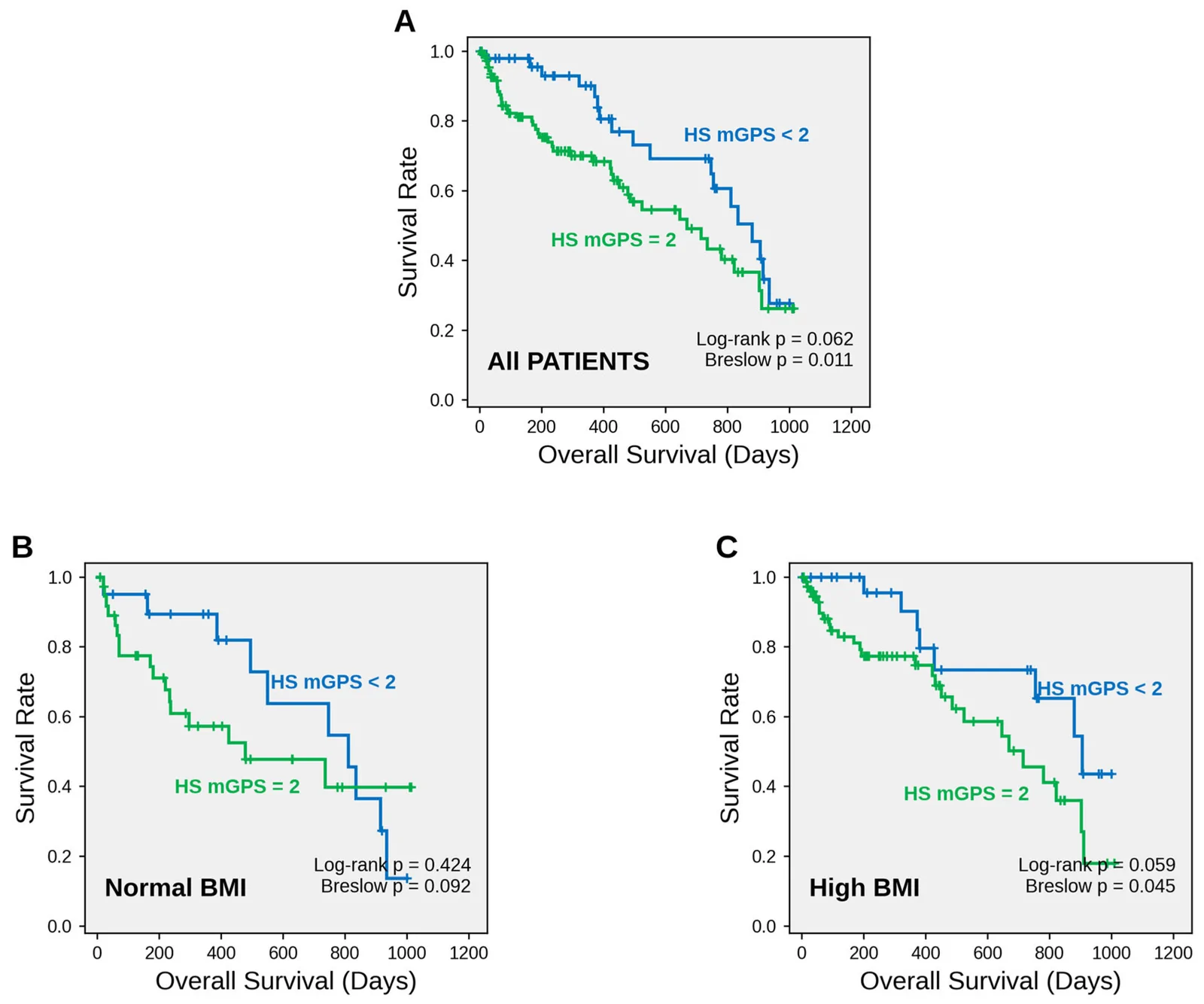
Kaplan–Meier overall survival (OS) curves stratified by the high-sensitivity modified Glasgow Prognostic Score (hs-mGPS; scores 0–1 vs. 2): (**A**) patients in the normal and high BMI groups combined (n = 163), (**B**) the normal BMI group (n = 58), and (**C**) the high BMI group (n = 105). *p*-values are from two-sided log-rank and Breslow tests.

Kaplan–Meier analyses showed that hs-mGPS was associated with overall survival (OS), with the difference concentrated in the earlier part of follow-up. Survival estimates for patients with hs-mGPS scores of 0 or 1 at 100, 300, 600, 900, and 1200 days were 97.9%, 92.9%, 69.2%, 45.4%, and 27.7%, respectively, compared with 82.2%, 69.9%, 54.5%, 36.6%, and 26.1% for those with hs-mGPS of 2 (Table S1). The difference was significant by the Breslow test, which weighs earlier events (*p* = 0.011), but not by the log-rank test (*p* = 0.062) (Figure 3A).

Stratified analyses by BMI showed differing patterns. In the normal BMI group, neither test reached statistical significance (Breslow *p* = 0.092; log-rank *p* = 0.424). In the high BMI group, the difference was significant by the Breslow test (*p* = 0.045) but not by the log-rank test (*p* = 0.059), again indicating a stronger influence on early survival (Figure 3B,C).

Within BMI strata, survival patterns diverged over time. In the normal BMI group, patients with hs-mGPS 0–1 had higher survival estimates through 600 days, after which the curves crossed (1200-day estimates, 13.7% vs. 39.8%). In the high BMI group, survival differences favored hs-mGPS 0–1 throughout follow-up (1200-day estimates, 43.5% vs. 18.0%).

### 3.3. Association Between Daily Weight Change by Percentile (DWtCP) and Overall Survival (OS)

**Figure 4:**
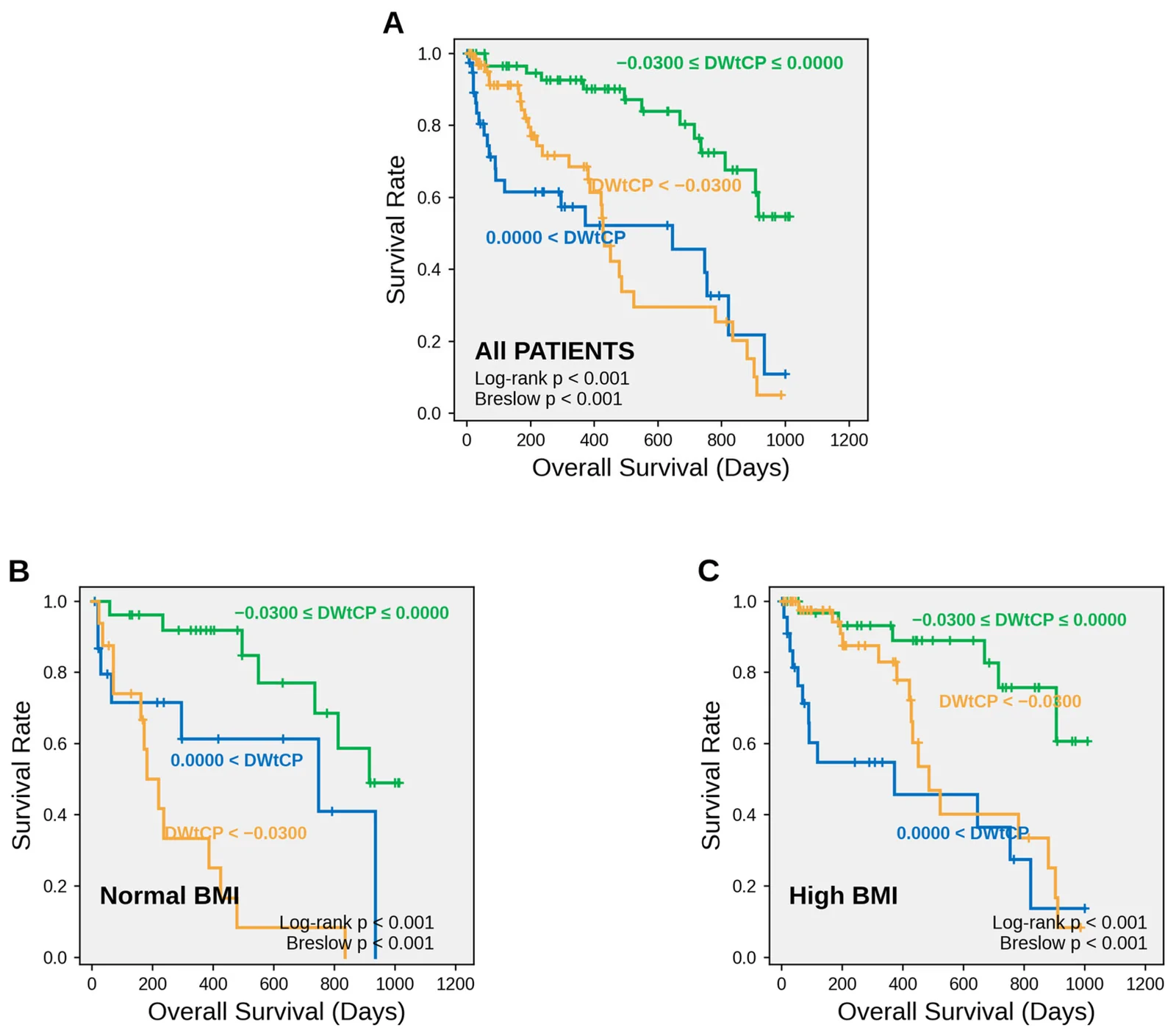
Kaplan–Meier overall survival (OS) curves by daily weight change by percentile (DWtCP) category (weight gain, moderate weight loss, and severe weight loss): (**A**) patients in the normal and high BMI groups combined (n = 163), (**B**) the normal BMI group (n = 58), and (**C**) the high BMI group (n = 105). *p*-values are from two-sided log-rank and Breslow tests.

Weight change, as measured by DWtCP, was strongly associated with OS (log-rank *p* < 0.001; Breslow *p* < 0.001; Figure 4A). Patients with moderate weight loss had the most favorable survival, with a 1200-day survival estimate of 54.6%, compared with 5.1% in the severe weight-loss group and 10.9% in the weight-gain group (Table S2). Pairwise comparison of the weight-gain and severe weight-loss groups showed a significant difference in early survival (Breslow *p* = 0.036) that was not sustained over time (log-rank *p* = 0.646).

BMI-stratified analyses confirmed the prognostic significance of DWtCP in both groups (normal BMI: log-rank *p* < 0.001, Breslow *p* < 0.001; high BMI: log-rank *p* < 0.001, Breslow *p* < 0.001; Figure 4B,C). In the normal BMI group, severe weight loss was associated with markedly poorer outcomes, with survival estimates of 8.3% at 600 days and 0% after 900 days, compared with 48.9% at 1200 days in the moderate weight-loss group. In the high BMI group, moderate weight loss remained associated with the best 1200-day survival estimate (60.6%), followed by weight gain (13.7%) and severe weight loss (8.4%).

### 3.4. Prognostic Factors Affecting Overall Survival (OS)

Univariate Cox regression analysis identified age, DWtCP, primary cancer site, albumin level, hs-CRP level, and hs-mGPS associated with OS at the screening threshold (two-sided *p* < 0.10). In multivariate analysis, DWtCP and primary cancer site remained independent predictors. Compared with weight gain, moderate weight loss was associated with significantly reduced mortality risk (HR = 0.194, 95% CI 0.093–0.405, *p* < 0.001). Relative to colorectal cancer, gastric (HR = 3.877, 95% CI 1.337–11.242, *p* = 0.013), pancreatic (HR = 3.705, 95% CI 1.706–8.047, *p* < 0.001), and hepatobiliary cancers (HR = 3.516, 95% CI 1.556–7.944, *p* = 0.003) were associated with increased mortality risk (Table 3).

BMI-stratified multivariate regression analyses revealed distinct prognostic profiles. In the normal BMI group, independent predictors of OS included DWtCP (moderate weight loss vs. weight gain: HR = 0.202, 95% CI 0.054–0.754, *p* = 0.017) and primary cancer site (pancreatic vs. colorectal: HR = 6.170, 95% CI 1.403–27.130, *p* = 0.016; hepatobiliary vs. colorectal: HR = 5.924, 95% CI 1.054–33.295, *p* = 0.043); hs-CRP was not individually significant (HR = 1.003, 95% CI 1.000–1.007, *p* = 0.092). In the high BMI group, independent predictors included DWtCP (moderate weight loss vs. weight gain: HR = 0.198, 95% CI 0.068–0.574, *p* = 0.003) and gastric cancer (HR = 4.571, 95% CI 1.144–18.257, *p* = 0.032); pancreatic (HR = 2.704, 95% CI 0.902–8.105, *p* = 0.076) and hepatobiliary cancers (HR = 2.845, 95% CI 0.953–8.496, *p* = 0.061) and albumin (HR = 0.467, 95% CI 0.204–1.067, *p* = 0.071) showed consistent directions of association but did not reach statistical significance (Table 3).

The proportional hazards assumption, assessed with scaled Schoenfeld residuals, was satisfied for most covariates but not for DWtCP (severe weight loss: *p* = 0.008 in the overall model and *p* = 0.005 in the high-BMI model; moderate weight loss: *p* = 0.024 in the normal-BMI model) or for hs-mGPS in the overall model (*p* = 0.018), indicating that these effects varied over follow-up. In a sensitivity analysis with follow-up divided at 300 days, corresponding to the median observation time, the survival advantage in the moderate weight-loss group compared to the weight-gain group was concentrated in the first 300 days (HR = 0.130, 95% CI 0.043–0.399) and attenuated thereafter (HR = 0.568, 95% CI 0.207–1.559); severe weight loss likewise showed a reduced early hazard (HR = 0.446, 95% CI 0.204–0.974) that did not persist in the later period (HR = 2.249, 95% CI 0.854–5.925). Period-specific estimates within BMI strata are provided in Supplement Table S3.

## 4. Discussion

This study demonstrates that longitudinal weight change and systemic inflammation are key determinants of overall survival in patients with GI cancers. Specifically, DWtCP and primary cancer site were significantly associated with survival, and hs-mGPS stratified early survival, with distinct patterns observed across BMI groups. Consistent with prior literature, increasing age was associated with poorer survival [20], particularly among patients with higher BMIs. This finding likely reflects the cumulative burden of comorbid conditions, including cardiovascular and metabolic disorders, which are more prevalent in older individuals with elevated BMI and may exacerbate cancer progression and limit treatment tolerance [21,22]. The interaction between age, adiposity, and systemic inflammation may further compound risk in this subgroup.

A notable finding was that moderate weight loss was associated with improved survival compared with both weight gain and severe weight loss, independent of BMI. This pattern was consistent across overall and stratified analyses and aligns with prior evidence suggesting that modest weight loss (<10%) may reflect less aggressive disease or preserved metabolic adaptability in patients with solid tumors [23]. In contrast, severe weight loss likely reflects advanced cachexia and catabolic dysregulation, which are well-established predictors of poor outcomes [24,25,26]. Importantly, the severe weight-loss category in our study (DWtCP < −0.03% per day) approximates the consensus cachexia threshold of >5% weight loss within 6 months [2], supporting the clinical relevance of this metric.

The observed association between weight gain and poorer survival warrants careful interpretation. Weight gain in this context may mask sarcopenia associated with loss of lean body mass, which is replaced by fluid retention or increased adiposity [27], both of which can occur in the setting of systemic inflammation and disease progression. As suggested in prior studies, sarcopenia may be masked by stable or increasing body weight, leading to an underestimation of nutritional and functional decline [27]. This mechanism may also help explain the survival patterns observed among patients with elevated hs-mGPS scores, particularly in the normal BMI group, where inflammatory processes and fluid shifts could transiently offset apparent weight loss [28].

Inflammation emerged as a central prognostic pathway in this study. Elevated hs-mGPS was associated with reduced early survival in unadjusted analyses. Although hs-CRP and albumin did not reach individual significance in BMI-stratified multivariate models, their directions of association were consistent, and the two markers together were more clinically informative. These findings reinforce the role of systemic inflammation as a driver of cancer progression and cachexia. In patients with higher BMI, the pro-inflammatory milieu associated with excess adipose tissue may further amplify this effect.

Adipose tissue-derived cytokines and the mobilization of free fatty acids during weight loss may contribute to tumor progression and metabolic dysregulation, particularly in obese patients [29]. The stronger and more consistent prognostic value of hs-mGPS in the high BMI group supports a biologically plausible interaction between adiposity and inflammation. Future studies should examine recent advances in cytokine profiles, such as IL-27, IL-33 (pro-inflammatory cytokines), IL-35, and IL-37 (anti-inflammatory cytokines), to clarify their roles in managing inflammation associated with cancer cachexia and potential therapeutic modalities [30,31,32,33,34].

Tumor site was also an independent predictor of survival, with pancreatic, gastric, and hepatobiliary cancers associated with significantly worse outcomes compared with colorectal cancer. These findings are consistent with established epidemiologic data demonstrating poorer prognosis and more aggressive disease trajectories in these malignancies [35]. Although cancer stage did not remain a significant predictor in multivariate models, this may reflect missing staging data and limited statistical power rather than a true lack of association.

It is well established that early detection of GI cancers leads to higher survival rates, as interventions can be implemented to slow or prevent disease progression [36]. However, this was not significant in this study. A higher risk of mortality is associated with the location of malignancy, as pancreatic and gastric cancer demonstrated lower survival in the normal and high BMI groups, respectively. Both pancreatic and gastric cancers are associated with lower survival six months post-diagnosis compared to colorectal cancer [37].

While the study has multiple strengths, it also has limitations. The study used retrospective data from a single institution in the Southeastern United States. The generalizability of the study findings is limited to the population in a similar area. Second, the inclusion criteria requiring available albumin and hs-CRP data may have introduced selection bias, as hs-CRP is more likely to be obtained in clinically complex or symptomatic patients. Third, the exclusion of patients with low BMI due to small sample size restricts interpretation across the full BMI spectrum, particularly given the known vulnerability of patients with low BMI for poor outcomes. Fourth, no treatment status or modality was available in the data for analysis. Thus, the results should be interpreted with caution, taking into account the limitations of the study. Fifth, analysis of individual biomarkers within strata has limitations due to modest subgroup sample sizes. Sixth, the period-specific hazard ratios for the later interval carry wide confidence intervals, which necessitate careful interpretation of the stratified and time-dependent results. Finally, the heterogeneity of GI cancers, including differences in tumor biology, stage, and anatomic factors that affect nutrient intake and absorption, may confound the observed relationships between weight change, inflammation, and survival.

Despite these limitations, this study provides clinically relevant insights into the complex interplay among weight dynamics, inflammation, and survival in GI cancer populations. The use of a longitudinal weight change metric (DWtCP) and the incorporation of hs-mGPS strengthen the ability to capture both the nutritional and inflammatory dimensions of prognosis.

## 5. Conclusions

This study highlights the prognostic significance of systemic inflammation and weight change in patients with GI cancers. The hs-mGPS was associated with overall survival in univariate analyses and demonstrated particular utility in stratifying risk among patients with higher BMIs. Moderate weight loss was consistently associated with improved survival. In contrast, both weight gain and severe weight loss were linked to poorer outcomes, underscoring the importance of a nuanced interpretation of weight trajectories in this population.

Albumin and hs-CRP, while individually informative, did not consistently differentiate survival risk across BMI groups, suggesting that composite indices such as hs-mGPS may provide greater clinical utility. These findings support the potential utility of hs-mGPS as a pragmatic, accessible adjunct for risk stratification, pending external validation in larger, prospective, and homogeneous GI cancer cohorts. Clinicians should consider longitudinal monitoring of inflammatory biomarkers and weight patterns from diagnosis through treatment. Such an approach may enhance early identification of high-risk patients, inform nutritional and supportive care interventions, and ultimately guide more individualized treatment strategies.

## Figures and Tables

**Table 1:** Scoring guidelines for GPS, mGPS, and hs-mGPS.

	Scores (Points)		
Variables	GPS	mGPS	hs-mGPS
CRP ≤ 10 mg/L & albumin ≥ 3.5 g/dL	0	0	
CRP ≤ 10 mg/L & albumin < 3.5 g/dL	1	0	
CRP > 10 mg/L & albumin ≥ 3.5 g/dL	1	1	
CRP > 10 mg/L & albumin < 3.5 g/dL	2	2	
hs-CRP ≤ 3 mg/L & albumin ≥ 3.5 g/dL			0
hs-CRP ≤ 3 mg/L & albumin < 3.5 g/dL			0
hs-CRP > 3 mg/L & albumin ≥ 3.5 g/dL			1
hs-CRP > 3 mg/L & albumin < 3.5 g/dL			2

GPS: Glasgow Prognostic Score; mGPS: modified GPS; hs-mGPS: high-sensitivity mGPS; CRP: C-reactive protein; hs-CRP: high-sensitivity CRP.

**Table 2:** Patient characteristics by BMI category.

Variables	All N (%)	Low BMI n_1_ (%)	Normal BMI n_2_ (%)	High BMI n_3_ (%)	*p* *
Total	176 (100)	13 (7.4)	58 (33.0)	105 (59.7)	
Ethnicity					0.156
Black	31 (17.6)	2 (15.4)	7 (12.1)	22 (21.0)	
White	145 (82.4)	11 (84.6)	51 (87.9)	83 (79.0)	
Gender					0.532
Male	104 (59.1)	6 (46.2)	33 (56.9)	65 (61.9)	
Female	72 (40.9)	7 (53.8)	25 (43.1)	40 (38.1)	
Age					0.102
<65 years	82 (46.6)	9 (69.2)	21 (36.2)	52 (49.5)	
≥65 years	94 (53.4)	4 (30.8)	37 (63.8)	53 (50.5)	
DWtCP					0.073 ^b^
Wt gain	41 (23.3)	2 (15.4)	16 (27.6)	23 (21.9)	
Mod. wt loss	67 (38.1)	7 (53.8)	26 (44.8)	34 (32.4)	
Severe wt loss	68 (38.6)	4 (30.8)	16 (27.6)	48 (45.7)	
Cancer Stage					0.360 ^b^
Stages 0 & 1	27 (15.3)	2 (15.4)	9 (15.5)	16 (15.2)	
Stage 2	38 (21.6)	3 (23.1)	9 (15.5)	26 (24.8)	
Stage 3	23 (13.1)	1 (7.7)	10 (17.2)	12 (11.4)	
Stage 4	38 (21.6)	5 (38.5)	15 (25.9)	18 (17.1)	
Unknown	47 (26.7)	1 (7.7)	14 (24.1)	32 (30.5)	
Missing	3 (1.7)	1 (7.7)	1 (1.7)	1 (1.0)	
Primary Cancer					0.412 ^b^
Esophageal Ca	19 (10.8)	1 (7.7)	7 (12.1)	11 (10.5)	
Gastric Ca	11 (6.3)	1 (7.7)	3 (5.2)	7 (6.7)	
Pancreatic Ca	51 (29.0)	7 (53.8)	20 (34.5)	24 (22.9)	
Hepatobiliary Ca	44 (25.0)	1 (7.7)	11 (19.0)	32 (30.5)	
Colorectal Ca	51 (29.0)	3 (23.1)	17 (29.3)	31 (29.5)	
Anemia					0.614
No	107 (60.8)	10 (76.9)	33 (56.9)	64 (61.0)	
Yes	69 (39.2)	3 (23.1)	25 (43.1)	41 (39.0)	
Biomarkers					0.284 ^c^
Albumin (g/dL) ^a^	3.00	3.00	3.10	2.90	
	(2.44–3.60)	(2.50–3.85)	(2.40–3.63)	(2.40–3.50)	
hs-CRP (mg/L) ^a^	70.20	85.90	45.00	91.80	0.014 ^c^
	(23.80–163.70)	(22.70–177.45)	(13.13–134.90)	(31.60–193.90)	
hs-mGPS					0.533 ^b^
0	8 (4.5)	0 (0.0)	4 (6.9)	4 (3.8)	
1	45 (25.6)	4 (30.8)	16 (27.6)	25 (23.8)	
2	123 (69.9)	9 (69.2)	38 (65.5)	76 (72.4)	

^a^ Median (IQR); ^b^ Fisher’s exact *p*-value; ^c^ Jonckheere–Terpstra Test *p*-value; *p* *: comparison of normal vs. high BMI groups; DWtCP = daily weight change by percentile; IQR = interquartile range; hs-CRP = high-sensitivity C-reactive protein; hs-mGPS = high-sensitivity modified Glasgow Prognostic Score; Wt: weight: Mod. weight loss= moderate weight loss (−0.03 ≤ DWtCP ≤ 0.00); severe weight loss = (DWtCP < −0.03).

**Table 3:** Prognostic factors affecting overall survival based on two BMI groups (excluding the low BMI group due to n = 13).

Variables	Normal BMI Group (n = 58)				High BMI Group (n = 105)			
	Univariate		Multivariate		Univariate		Multivariate	
	HR (95%CI)	*p*	HR (95%CI)	*p*	HR (95%CI)	*p*	HR (95%CI)	*p*
Ethnicity								
Black	1				1			
White	0.519 (0.205–1.314)	0.166			1.275 (0.555–2.929)	0.567		
Gender								
Male	1				1			
Female	1.196 (0.560–2.554)	0.644			1.006 (0.514–1.967)	0.986		
Age								
<65 years	1				1		1	
≥65 years	1.179 (0.535–2.599)	0.683			2.057 (1.043–4.056)	0.037	1.437 (0.650–3.173)	0.370
DWtCP								
Weight gain	1		1		1		1	
Moderate weight loss	0.324 (0.113–0.931)	0.036	0.202 (0.054–0.754)	0.017	0.154 (0.058–0.407)	<0.001	0.198 (0.068–0.574)	0.003
Severe weight loss	2.643 (1.012–6.900)	0.047	1.540 (0.442–5.365)	0.498	0.522 (0.250–1.091)	0.084	0.477 (0.188–1.214)	0.120
Cancer Stage								
Stages 0 & 1	1		1		1			
Stage 2	0.107 (0.013–0.911)	0.039	0.453 (0.040–5.117)	0.522	1.647 (0.513–5.285)	0.402		
Stage 3	0.353 (0.102–1.223)	0.100	2.094 (0.414–10.602)	0.372	1.728 (0.462–6.464)	0.416		
Stage 4	0.467 (0.156–1.402)	0.174	1.120 (0.269–4.664)	0.876	1.442 (0.406–5.127)	0.572		
Unknown	0.374 (0.115–1.217)	0.102	2.048 (0.335–12.523)	0.438	2.098 (0.654–6.732)	0.213		
Primary Cancer Site								
Esophageal Ca	3.222 (0.802–12.946)	0.099	2.552 (0.509–12.785)	0.255	0.800 (0.170–3.773)	0.778	0.821 (0.157–4.299)	0.815
Gastric Ca	5.981 (1.053–33.978)	0.044	2.187 (0.276–17.312)	0.459	5.702 (1.637–19.855)	0.006	4.571 (1.144–18.257)	0.032
Pancreatic Ca	4.999 (1.570–15.919)	0.006	6.170 (1.403–27.130)	0.016	2.519 (0.898–7.064)	0.079	2.704 (0.902–8.105)	0.076
Hepatobiliary Ca	3.878 (0.997–15.077)	0.050	5.924 (1.054–33.295)	0.043	3.263 (1.319–8.074)	0.011	2.845 (0.953–8.496)	0.061
Colorectal Ca	1		1		1		1	
Anemia								
No	1				1			
Yes	1.183 (0.553–2.531)	0.665			1.458 (0.743–2.861)	0.273		
Biomarkers, median (IQR)								
Albumin	0.704 (0.429–1.156)	0.165			0.581 (0.379–0.893)	0.013	0.467 (0.204–1.067)	0.071
hs-CRP	1.002 (1.000–1.005)	0.116	1.003 (1.000–1.007)	0.092	1.002 (0.999–1.005)	0.191		
hs-mGPS								
0 & 1	1				1		1	
2	1.380 (0.624–3.052)	0.426			2.127 (0.956–4.734)	0.064	0.534 (0.110–2.603)	0.437

## Data Availability

The data analyzed during the current study are available from the corresponding author upon reasonable request.

## Supplementary Materials

The following supporting information can be downloaded at https://www.mdpi.com/article/10.3390/cancers18172808/s1, Table S1: OS rates for 100-, 300-, 600-, 900-, and 1200 days according to hs-mGPS in all patients, normal BMI group, and high BMI group; Table S2: OS rates for 100-, 300-, 600-, 900-, and 1200 days according to DWtCP in all patients, normal BMI group, and high BMI group; Table S3: Period-specific hazard ratios for DWtCP.

## Abbreviations

The following abbreviations are used in this manuscript:

AUC	area under the curve
BMI	body mass index
CI	confidence interval
CRP	C-reactive protein
CTSI	Clinical Translational Science Institute
DWtCP	daily weight change by percentile
GI	gastrointestinal
GLIM	Global Leadership Initiative on Malnutrition
GPS	Glasgow Prognostic Score
HR	hazard ratio
hs-CRP	high-sensitivity C-reactive protein
hs-mGPS	high-sensitivity modified Glasgow Prognostic Score
ICD	International Classification of Diseases
IDR	Integrated Data Repository
IL	interleukin
IQR	interquartile range
IRB	Institutional Review Board
mGPS	modified Glasgow Prognostic Score
OS	overall survival
SD	standard deviation
