# Supplementary material for: Integrating Systemic Inflammation and Longitudinal Weight Trajectories Improves Prognostic Stratification in Gastrointestinal Cancers: A Real-World Retrospective Study
Source: Cancers (Basel). 2026 Aug 29;18(17):2808. doi: 10.3390/cancers18172808 (PMC13565716; doi:10.3390/cancers18172808)
Supplement: Supplementary file 1 [file cancers-18-02808-s001.zip › cancers-4518549-supplementary.pdf]

Supplement Table S1. Kaplan–Meier overall survival estimates (%) and numbers at risk by hs-mGPS.

Group	Category (n)	100 d	300 d	600 d	900 d	1200 d	Breslow *p*	Log-rank *p*
All patients	hs-mGPS 0–1 (49)	97.9	92.9	69.2	45.4	27.7	.011	.062
	No. at risk	*43*	*32*	*18*	*9*	*0*		
	hs-mGPS 2 (114)	82.2	69.9	54.5	36.6	26.1		
	No. at risk	*75*	*47*	*23*	*7*	*0*		
Normal BMI	hs-mGPS 0–1 (20)	95.0	89.4	63.7	36.4	13.7	.092	.424
	No. at risk	*18*	*14*	*7*	*4*	*0*		
	hs-mGPS 2 (38)	77.5	57.3	47.7	39.8	39.8		
	No. at risk	*27*	*15*	*8*	*3*	*0*		
High BMI	hs-mGPS 0–1 (29)	100.0	95.5	73.4	54.4	43.5	.045	.059
	No. at risk	*25*	*18*	*11*	*5*	*0*		
	hs-mGPS 2 (76)	84.6	77.3	58.5	35.9	18.0		
	No. at risk	*48*	*32*	*15*	*4*	*0*		

All tests are two-sided. No patient remained under observation beyond 1,013 days; estimates at 1200 days correspond to the final plateau of the survival curve.

Supplement Table S2. Kaplan–Meier overall survival estimates (%) and numbers at risk by DWtCP.

Group	Category (n)	100 d	300 d	600 d	900 d	1200 d	Log-rank *p*
All patients	Weight gain (39)	64.7	57.4	52.2	21.7	10.9	<.001
	No. at risk	*20*	*13*	*9*	*2*	*0*	
	Moderate loss (60)	96.4	92.5	83.9	67.6	54.6	
	No. at risk	*54*	*43*	*25*	*11*	*0*	
	Severe loss (64)	91.2	71.6	29.6	15.2	5.1	
	No. at risk	*44*	*23*	*7*	*3*	*0*	
Normal BMI	Weight gain (16)	71.5	61.3	61.3	40.9	0.0	<.001
	No. at risk	*9*	*5*	*4*	*1*	*0*	
	Moderate loss (26)	96.2	91.8	77.0	58.7	48.9	
	No. at risk	*25*	*20*	*10*	*6*	*0*	
	Severe loss (16)	74.0	33.3	8.3	0.0	0.0	
	No. at risk	*11*	*4*	*1*	*0*	*0*	
High BMI	Weight gain (23)	60.2	54.7	45.6	13.7	13.7	<.001
	No. at risk	*11*	*8*	*5*	*1*	*0*	
	Moderate loss (34)	96.7	93.2	89.0	75.7	60.6	
	No. at risk	*29*	*23*	*15*	*5*	*0*	
	Severe loss (48)	97.5	87.5	40.1	25.1	8.4	
	No. at risk	*33*	*19*	*6*	*3*	*0*	

Breslow $p<.001$ for all three group comparisons. All tests are two-sided. No patient remained under observation beyond 1,013 days.

Supplement Table S3. Period-specific hazard ratios for DWtCP (reference: weight gain), with follow-up divided at 300 days (median observation time, 292 days).

Model	DWtCP category	≤300 days, HR (95% CI)	>300 days, HR (95% CI)
All patients	Moderate loss	0.130 (0.043–0.399)	0.568 (0.207–1.559)
	Severe loss	0.446 (0.204–0.974)	2.249 (0.854–5.925)
Normal BMI	Moderate loss	0.165 (0.031–0.866)	0.516 (0.092–2.890)
	Severe loss	1.478 (0.469–4.660)	2.673 (0.359–19.894)
High BMI	Moderate loss	0.136 (0.028–0.657)	0.483 (0.107–2.182)
	Severe loss	0.160 (0.046–0.554)	1.996 (0.481–8.279)

Models are adjusted as in the corresponding main models (all patients and high BMI: age, primary cancer site, albumin, hs-mGPS; normal BMI: primary cancer site). Estimates for the later period rest on small numbers at risk and carry wide confidence intervals.
